# A Case of Fungal Maxillary Sinusitis Extending to the Contralateral Side Through the Nasal Septum

**DOI:** 10.7759/cureus.39548

**Published:** 2023-05-26

**Authors:** Naoyuki Matsumoto, Ryoji Kagoya, Mariko Yasui, Hiroshi Uozaki, Ken Ito

**Affiliations:** 1 Otolaryngology, Teikyo University, Tokyo, JPN; 2 Otorhinolaryngology-Head and Neck Surgery, The University of Tokyo, Tokyo, JPN; 3 Pathology, Teikyo University, Tokyo, JPN

**Keywords:** bone destruction, osteoporosis, nasal septum, contralateral extension, fungal rhinosinusitis

## Abstract

Fungal rhinosinusitis (FRS) presents as various phenotypes ranging from asymptomatic colonization to life-threatening infections. Here, we report an atypical case of FRS of the left maxillary sinus that extended to the contralateral maxillary sinus through the nasal septum. An 80-year-old woman with a history of osteoporosis was referred to our hospital for further management of headaches and chronic rhinosinusitis. Computed tomography (CT) of the sinus revealed a mass lesion with calcification in the left maxillary sinus, extending to the contralateral maxillary sinus through the nasal septum. T1-weighted and T2-weighted magnetic resonance imaging revealed a mass lesion with low-intensity signals. Endoscopic sinus surgery was performed for the diagnosis and treatment. Histopathological examination revealed fungal elements in the caseous material of the left maxillary sinus. However, no tissue-invasive fungal forms were found. Additionally, eosinophilic mucin was not observed. Based on these findings, the patient was diagnosed with fungus ball (FB). To the best of our knowledge, there are no reports of a FB extending contralaterally through the nasal septum. This report serves as a reminder that FB can extend into contralateral paranasal sinuses through the nasal septum and the possibility that osteoporosis is a cause of extensive bone destruction.

## Introduction

Osteolytic lesions in the nasal cavity and paranasal sinuses should be carefully investigated to exclude the possibility of malignancy. Additionally, it is important to note that fungal rhinosinusitis (FRS) can cause bone destruction and remodeling. FRS represents a spectrum of diseases ranging from asymptomatic colonization to life-threatening infections [1]. Generally, FRS is divided into two subgroups, based on the presence or absence of fungal hyphae within the mucosa or the bone of the sinuses: invasive FRS (IFRS) and non-invasive FRS, respectively [2]. IFRS is further subdivided into acute, chronic, and chronic granulomatous types. Among them, acute IFRS is the most fatal disease, with a reported mortality rate of 50% [3]. IFRS is commonly accompanied by intraorbital, intracranial, or maxillofacial extension. In contrast, non-invasive FRS is an indolent disease and has a good prognosis, although there have been a few cases of IFRS developing from non-invasive FRS in one to several years [4-6]. Non-invasive FRS is subdivided into allergic FRS (AFRS) and fungus ball (FB) [7]. However, the bony wall of the paranasal sinuses sometimes expands and is destroyed because of pressure necrosis. Bone erosion is observed in 4%−17% of FB cases [2,8]. In addition, 6-56% of patients with AFRS have an expanding mass that leads to bony erosion [9]. Here, we report a rare case of non-invasive FRS that originated from the left maxillary sinus and extended to the contralateral nasal cavity and maxillary sinus through the nasal septum.

## Case presentation

**Figure 1:**
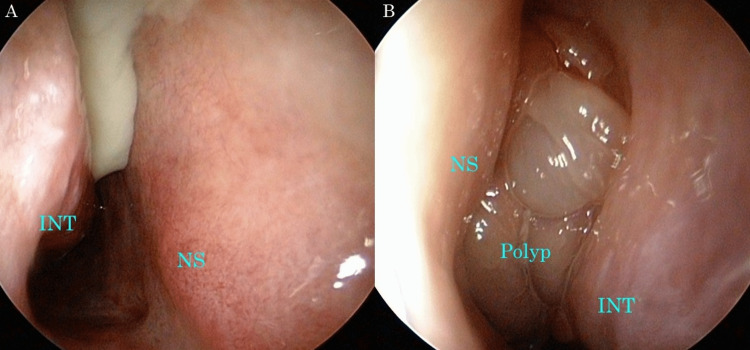
Images of nasal endoscopy at first visit (A) Endoscopic image of the right nasal cavity. Purulent discharge is observed. (B) Endoscopic image of the left nasal cavity. A polypoid mass with smooth surface is occupying the common nasal meatus. INT: inferior nasal turbinate, NS: nasal septum

An 80-year-old woman with a history of osteoporosis, hypertension, and depression was diagnosed with chronic rhinosinusitis (CRS) by computed tomography (CT) of the head at a local hospital after a thorough evaluation of her persistent headache and bilateral nasal congestion for several years. Her medications were alprazolam, paroxetine, and ramelteon for depression. She had no history of sinonasal surgery or trauma. She received low-dose macrolide therapy for two months. As the symptoms did not improve, she was referred to our hospital for further treatment. Nasal endoscopy revealed a polypoid mass with smooth surface occupying the left common nasal meatus and purulent discharge in the right common nasal meatus (Figure 1). A biopsy of the polypoid mass in the left nasal cavity did not reveal any evidence of malignancy. Blood test results showed normal 1,3-β-D-glucan levels, normal white blood cell (WBC) count with normal eosinophil count, normal total immunoglobulin (Ig) E level, and positivity for anti-Aspergillus IgE antibody (Table 1).

**Table 1:** Blood test results WBC: white blood cell; Ig: immunoglobulin

Item	Result	Reference range
WBC (/μl)	5800	3300–8600
Eosinophil (%)	1	0–2
1,3-β-D-glucan (pg/mL)	<6.0	<6.0
Total IgE (IU/mL)	108	0–170
Anti-Aspergillus IgE	+	-

**Figure 2:**
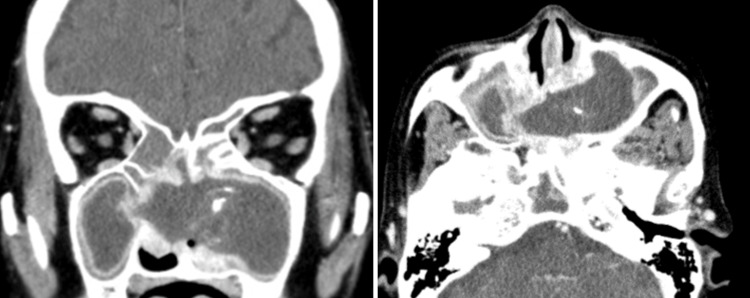
Contrast-enhanced sinus CT images Coronal and axial images show a mass lesion with enhanced margins and central calcification in the left maxillary sinus, extending to the contralateral maxillary sinus through the nasal septum. CT: computed tomography

**Figure 3:**
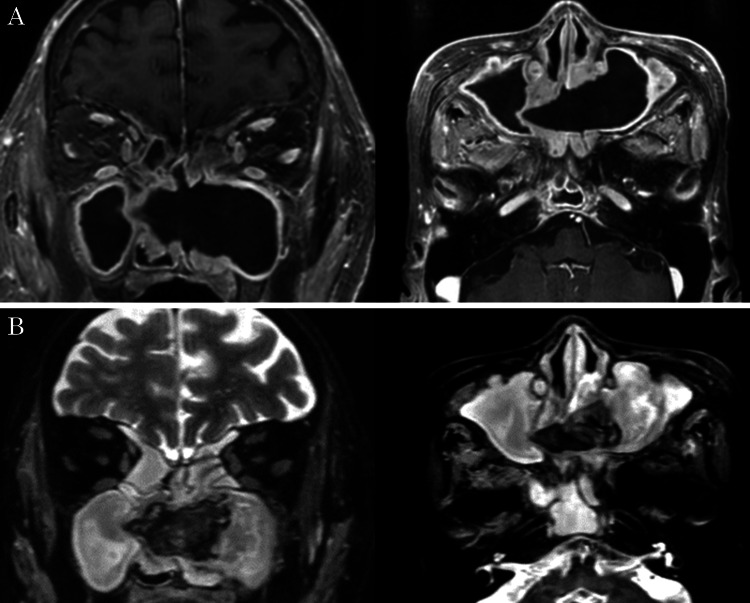
Gadolinium-enhanced sinus MRI (A) T1-weighted and (B) T2-weighted images show a mass lesion with low-intensity signals in the left maxillary sinus extending to the contralateral maxillary sinus through the nasal septum MRI: magnetic resonance image

Contrast-enhanced CT of the sinus revealed a mass lesion with calcification in the left maxillary sinus extending to the contralateral maxillary sinus through the nasal septum (Figure 2). The nasal septum defect was located in the vomer and the perpendicular plate of the ethmoid bone, with a diameter of 24 mm in the anteroposterior direction and 30 mm in the craniocaudal direction. To facilitate a further examination of the mass, gadolinium-enhanced magnetic resonance imaging (MRI) of the sinus was performed on the patient. T1-weighted and T2-weighted MRI showed a mass lesion with low-intensity signals (Figure 3). Based on these findings, non-invasive FRS was suspected. As the patient tested positive for anti-Aspergillus IgE antibody, there was a possibility of AFRS.

**Figure 4:**
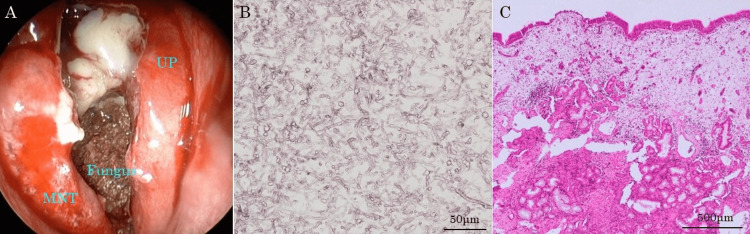
Intraoperative and histopathological images (A) Intraoperative endoscopic findings of the left middle meatus. Ceasus material and purulent discharge are confirmed. (B) Grocott-stained section of the ceasus material. Fungal elements with Y-shaped branches are confirmed. (C) Hematoxylin and eosin-stained section of the left maxillary sinus mucosa as a representative. No tissue-invasive fungal forms were observed. MNT: middle nasal turbinate, UP: uncinate process

**Figure 5:**
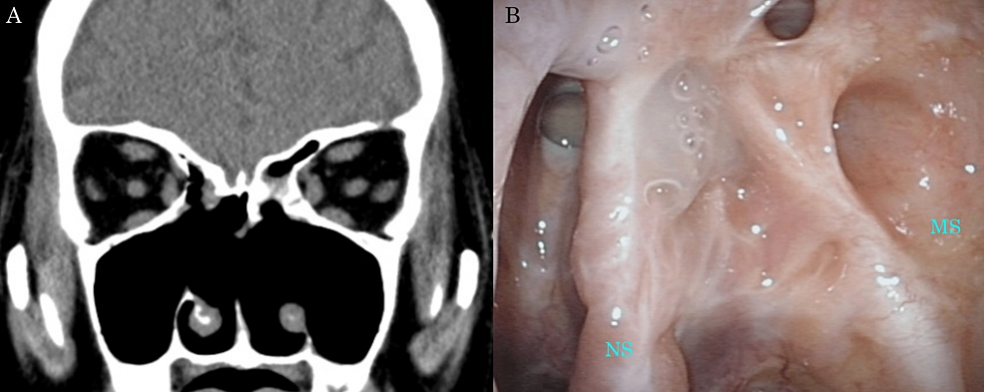
Images of sinus CT and nasal endoscopy one year after surgery (A) Sinus CT and (B) nasal endoscopy revealed no recurrence of FRS. CT: computed tomography, FRS: fungal rhinosinusitis, MS: maxillary sinus, NS: nasal septum

The patient underwent endoscopic sinus surgery (ESS) for diagnosis and treatment. Intraoperatively, a caseous mass accompanied by purulent discharge extending from the left maxillary sinus to the contralateral maxillary sinus was observed (Figure 4A). There was partial destruction of the vomer and the perpendicular plate, but no defect of nasal septal cartilage was observed. The mucosa of the maxillary sinus and ethmoid sinus was edematous and thickened on both sides. Biopsy from bilateral maxillary and ethmoid sinus mucosa was performed, considering the possibility of IFRS and neoplastic lesions. Allergic mucins were not detected in the paranasal sinuses. Histopathological examination revealed fungal elements with Y-shaped branches within the caseous material (Figure 4B). No tissue-invasive fungal forms were observed in all biopsied mucosal tissues (Figure 4C). Additionally, there was no significant eosinophil infiltration within the sinus mucosal tissue. FB was diagnosed based on the histopathological results. At the one-year follow-up, no recurrence was observed in the patient without antifungal treatment (Figure 5A, 5B).

## Discussion

Approximately 10% of the patients who undergo ESS are diagnosed with FRS. In more than 90% of the FRS cases, a single sinus is involved, and unilateral involvement is present in almost 99% of the cases [8]. The most commonly involved sinus (94%) is the maxillary sinus [9]. In patients with FRS, the bony walls of the paranasal sinuses usually become sclerotic and thickened because of chronic inflammation. However, they sometimes undergo thinning and destruction because of pressure necrosis [2,8]. In cases of AFRS, expanding mucin exerts pressure on the mucosal blood vessels and compromises the blood supply to the underlying bone. Consequently, the bony structures are susceptible to mechanical stress and necrosis [10]. According to Kim et al. [11] the sphenoid sinus exhibited a higher incidence of bone-eroding FB than the maxillary sinus. They reported that the FB of the maxillary sinus extended to the orbit, infratemporal fossa, and cheek. AlQahtani et al. [12] reported a case of AFRS that recurred with contralateral sinus involvement after surgery.

To the best of our knowledge, there are no reports of an FB originating from the maxillary sinus and extending to the contralateral maxillary sinus through the nasal septum. Contralateral involvement in non-invasive FRS is extremely rare. In the present case, the preoperative blood test showed a positive result for Aspergillus fumigatus-specific IgE, whereas eosinophilic mucin was not observed in the paranasal sinuses. Therefore, the patient was diagnosed with FB. Considering that reported cases of FB progressed to the IFRS over a period of one to several years [5,6], the present case may extend contralaterally over several years.

We need to note that the patients also had osteoporosis. Some reports have described the relationship between osteoporosis and paranasal sinuses. Choi et al. [13] reported that the prevalence of CRS was 1.5-fold higher in patients with osteoporosis than in healthy individuals. Lee et al. [14] revealed using a CT scan that the density of the midfacial bone was reduced in patients with osteoporosis compared to that in controls. In the present case, there is a possibility that osteoporosis had an impact on the vulnerability of the bone of the nasal septum. The exact reason why the FB spread contralaterally rather than laterally to the maxillary sinus remains unclear. It is speculated that the presence of a natural foramen of the maxillary sinus and the thinness of the nasal septal bone compared to the maxillary bone are factors.

## Conclusions

We report a rare case of FRS that extended to the contralateral side through the nasal septum. The present case adds to the current knowledge by emphasizing that non-invasive FRS can cause extensive bone destruction, especially in the presence of osteoporosis is present.
